# Systemic Effects of Enzymatic Necrosectomy in Minor Burn Wounds Using NexoBrid^®^

**DOI:** 10.3390/jpm15080330

**Published:** 2025-07-25

**Authors:** David Breidung, Moritz Billner, Felix Ortner, Philipp von Imhoff, Simonas Lapinskas, Konrad Karcz, Sarina Delavari, Denis Ehrl

**Affiliations:** Department of Plastic, Reconstructive and Hand Surgery, Burn Unit, Paracelsus Medical University, Klinikum Nürnberg, Breslauer Str. 201, 90471 Nuremberg, Germanyphilipp.vonimhoff@klinikum-nuernberg.de (P.v.I.); simonas.lapinskas@klinikum-nuernberg.de (S.L.); konrad.karcz@klinikum-nuernberg.de (K.K.); sarina.delavari@klinikum-nuernberg.de (S.D.); denis.ehrl@klinikum-nuernberg.de (D.E.)

**Keywords:** enzymatic debridement, burn injuries, systemic inflammatory response, NexoBrid, Bromelain

## Abstract

**Background/Objectives:** Enzymatic debridement with NexoBrid^®^ is an effective alternative to surgical debridement in burn care, but its potential systemic effects remain unclear. In the context of personalized burn care, understanding individual patient responses to topical agents is essential to optimize outcomes and minimize risks. This study aimed to characterize laboratory and clinical parameter changes following NexoBrid^®^ application in patients with small burn injuries (≤10% TBSA). **Methods:** We retrospectively analyzed 75 burn patients treated with NexoBrid^®^ to evaluate changes in systemic inflammatory markers, coagulation parameters, and clinical parameters before and after enzymatic debridement. **Results:** Statistically significant increases in body temperature (*p* = 0.018), decreases in hemoglobin (*p* < 0.001), and increases in C-reactive protein (CRP) levels (*p* < 0.001) were observed, suggesting mild systemic inflammatory changes. However, leukocyte counts did not change significantly (*p* = 0.927), and body temperature remained within the normothermic range, indicating that these changes were not clinically significant. A significant decrease in the prothrombin time ratio (% of normal; *p* = 0.002) was also observed, suggesting potential impacts on coagulation. Importantly, while body temperature was slightly higher in patients with a higher degree of BSA exposure within the ≤10% TBSA cohort (*p* = 0.036), the extent of NexoBrid^®^ application did not correlate with other inflammatory markers. **Conclusions:** These findings suggest that measurable systemic changes can occur following NexoBrid^®^ application in small burns, particularly affecting inflammatory and coagulation parameters. These observations contribute to the understanding of treatment-related responses and may help inform clinical decision-making.

## 1. Introduction

Burn injuries are a major health concern worldwide, affecting millions of people every year and leading to significant challenges in both immediate and long-term care [1]. One of the key steps in treating burn wounds is debridement, which involves removing dead or damaged tissue to prevent infection and help the wound heal properly [2]. Formally, this has been achieved through surgical methods, which, while effective, can also be quite invasive. Surgical debridement often requires general anesthesia, especially for larger burns, and can result in the removal of healthy tissue along with the necrotic tissue or incomplete debridement on the other side [2]. While adequate analgesia, and in some cases general anesthesia, is also required for enzymatic debridement, the potential to avoid skin grafts means that local anesthesia can be a viable option in selected cases.

Since the European approval of NexoBrid^®^ in 2013, enzymatic debridement has emerged as a less invasive alternative [3]. This method uses enzymes to break down and remove dead tissue selectively, leaving healthy tissue intact [4]. Among the enzymatic agents available, NexoBrid^®^ (MediWound Ltd., Yavne, Israel) has become particularly well-known. The debridement agent is applied as a four-hour topical enzymatic treatment, intended for use by healthcare professionals in specialized burn care settings and recommended only for limited burn surface areas (10% in children and 15% in adults of total body surface area; TBSA) [5]. NexoBrid^®^ is made from bromelain, a group of enzymes derived from pineapple stems, which effectively targets and breaks down the proteins in burn eschar [6]. Studies have shown that using NexoBrid^®^ can speed up the process of debridement and reduce the skin graft area [6,7,8]. A recent multicenter randomized controlled trial demonstrated that NexoBrid^®^ is a safe and effective enzymatic debridement option for pediatric burn patients [9].

In addition to these benefits, NexoBrid^®^ has been linked to improved outcomes in terms of wound bed quality [10], which is crucial for successful skin grafting and reducing the risk of infection. Patients treated with NexoBrid^®^ often experience shorter hospital stays, which can lower overall healthcare costs and thereby improve their quality of life [11]. Enzymatic debridement has also gained recognition as an alternative to surgical debridement, especially in areas requiring functional preservation, such as the hands [12,13]. However, while the local effects of NexoBrid^®^ on the wound itself have been well-studied, there is still a lot we do not know about its effects on the body in its entirety.

As is well-known, burn injuries trigger a complex systemic response, including inflammation, immune system activation, and metabolic changes, which can sometimes lead to serious complications like sepsis or organ failure [1,14,15,16]. Although NexoBrid^®^ is applied directly to the wound, it is important to understand whether its components, like bromelain, could be absorbed into the bloodstream and potentially affect this systemic response. There are also concerns about allergic reactions or effects on blood clotting that need to be carefully considered [17,18,19].

In this study, our objective was to explore whether enzymatic debridement with NexoBrid^®^ in patients with ≤10% TBSA burns is associated with measurable changes in laboratory and clinical parameters. Understanding individual patient variability in terms of systemic response may contribute to risk stratification and tailored therapeutic decisions in the context of personalized burn care. Specifically, we aimed to characterize systemic inflammatory markers, metabolic parameters, and coagulation profiles before and after treatment.

This observational pre–post analysis aimed to characterize the systemic effects associated with enzymatic debridement using NexoBrid^®^ in minor burns, with a particular focus on inflammatory markers and coagulation parameters under routine clinical conditions.

## 2. Materials and Methods

We conducted a retrospective study to evaluate the systemic effects of enzymatic debridement using NexoBrid^®^ in patients with burn injuries. This study included cases from the Department of Plastic, Reconstructive and Hand Surgery, Burn Unit at Klinikum Nürnberg (Nuremberg, Germany). This research adhered to the ethical principles outlined in the Declaration of Helsinki. The study was registered at our study coordination centre in the hospital. As this was a retrospective analysis using fully anonymized data without patient contact or intervention, formal ethical approval was not required according to local institutional and regional guidelines (Bavarian Medical Law).

Data were obtained from our hospital databases, covering the period from January 2018 to March 2024. Patients included in the study had burn injuries treated with NexoBrid^®^ for enzymatic debridement. Cases were identified from our hospital database using the operation and procedure (OPS) code for occlusive dressing with enzymatic wound debridement for burns (8-191.7). Subsequently, a review of the surgical treatment was conducted using operating theater reports and doctors’ letters. Patients under the age of 18 and patients who had undergone conventional surgical necrosectomy before enzymatic debridement or in the same operation were excluded from the study. To limit the pathophysiological effects of the burn wounds on clinical and laboratory parameters, severely burned patients with an affected body surface area of 10% or more (according to the S2k guideline in Germany) were excluded from the study [20]. There were no additional exclusion criteria for the formation of the study population.

In our burn unit, patients with ≤10% TBSA burns are managed according to a structured clinical algorithm adapted to burn depth, localization, and extent. The following section outlines the relevant steps applied in this subgroup. Inhalation injuries were confirmed bronchoscopically in clinically suspected cases. Standard supportive treatment included oxygen therapy, secretion management, protective mechanical ventilation, and empiric antibiotic treatment when indicated. Upon admission, all patients underwent hydrotherapy to cleanse the burn wounds and facilitate an initial assessment of wound severity and condition. Subsequently, the burn wounds were evaluated to determine the presence of deep second-degree or third-degree circumferential burns. If such wounds were absent, Polyhexanide Gel Wound Dressings or Polyhexanide Fluid Moist Wound Dressings were applied in preparation for enzymatic debridement. Within 36 to 48 h post-burn, Laser Doppler Imaging was performed to assess burn depth accurately. Third-degree burns were identified based on clinical findings and supported by Laser Doppler Imaging when available. However, the precise percentage of third-degree TBSA was not routinely documented and was therefore not separately analyzed in this study. Based on wound progression and clinical judgment, early necrosectomy was then conducted.

In cases where deep second-degree or third-degree circumferential burns were present, wound localization was assessed to guide further treatment. For burns localized on the extremities, enzymatic debridement with NexoBrid^®^ was performed on the day of admission. Following debridement, Polyhexanide Fluid Moist Wound Dressings were applied for post-soaking care, and early necrosectomy was subsequently conducted. For burns located on the neck, thoracic wall, or abdomen, escharotomy was performed as the initial surgical intervention, followed by early necrosectomy.

The approach to early necrosectomy varied based on the depth and extent of the burn wounds. For superficial second-degree burns, a skin substitute (SUPRATHEL^®^) was applied to facilitate healing. In deep second-degree burns, enzymatic necrosectomy or hydrosurgery with VERSAJET was performed based on clinical judgment, considering factors such as eschar consistency, burn mechanism, and affected body region. Post-debridement, wound coverage was achieved using a skin substitute (SUPRA SDRM^®^ or Kerecis™). In cases where fatty tissue was exposed, split-thickness skin grafting was conducted.

In the case of third-degree burns, epifascial necrosectomy was performed. For smaller burn areas, split-thickness skin grafts were directly applied. In contrast, larger burn areas were initially treated with a skin substitute (NovoSorb^®^ Biodegradable Temporising Matrix) or managed with vacuum-assisted closure therapy to promote granulation tissue formation. Once sufficient granulation was observed, split-thickness skin grafting was performed to ensure optimal wound closure.

It is important to note that while this algorithm provided a structured framework for treatment, individual clinical judgment was applied, and deviations from the algorithm were made when deemed necessary to accommodate for patient-specific conditions and optimize outcomes.

We collected data on demographic characteristics, burn severity, and treatment details, including the percentage of the body surface area (BSA) affected and the extent of enzymatic debridement. We also recorded outcomes such as length of hospital stay, mortality rates, and postoperative complications. Pain was assessed using a numerical rating scale (NRS) ranging from 0 (no pain) to 10 (worst pain) as part of routine clinical monitoring, including during the period before and after the NexoBrid^®^ application. Upon admission and prior to hydrotherapy, swabs were taken for microbiological surveillance. During inpatient treatment, wound swabs were repeated as needed. If respiratory infection was suspected, tracheal secretions were sent for culture. Inflammatory markers were routinely monitored, and antibiotic therapy was initiated in coordination with the institutional antimicrobial stewardship team.

Laboratory parameters such as leucocyte count, hemoglobin levels, prothrombin time ratio (% of normal), activated partial thromboplastin time, sodium, potassium, creatinine, and C-reactive protein (CRP) levels were assessed within 24 h before and within 24 h after the application of NexoBrid^®^ to evaluate potential systemic effects. In addition, the results were compared between patients with limited use of NexoBrid^®^ (less than 3% BSA treated) and those with a higher degree of use (more than 3% BSA). The 3% threshold was chosen because it allowed for a meaningful stratification of the cohort based on treatment extent and corresponded to a practical distinction in documentation via procedural codes. The exact sampling times varied due to the retrospective nature of the data collection and routine ward-based clinical practice. The NexoBrid^®^ application protocol followed the manufacturer’s instructions: the enzymatic agent was applied to the burn wound and left in place for four hours before removal. The dosage was determined according to the wound size as per the product’s guidelines. Anesthesia during NexoBrid^®^ treatment was tailored to the location and size of the burn area: smaller areas were managed with local infiltration anesthesia, while larger or more sensitive areas (e.g., hands, face) were treated under regional anesthesia (plexus block) or sedation/general anesthesia, as clinically indicated. No imputation was performed for missing data; only cases with complete pre–post data for the analyzed parameters were included in the respective statistical analyses.

Data collection was executed in Microsoft^®^ Excel (Version 16.98, Microsoft Corporation, Redmond, WA, USA) and statistical analysis was conducted in Microsoft^®^ Excel and IBM^®^ SPSS^®^ Statistics (Version 31, IBM Corp, Armonk, NY, USA). Significances were analyzed using paired *t*-tests for pre–post comparisons within the same patients, and unpaired *t*-tests (independent *t*-tests) were used for comparisons between different groups (e.g., higher vs. lower degree of BSA exposure). Chi-squared tests were used for categorical variables, and a two-sided *p*-value < 0.05 was set as the significance level. Categorical variables were reported as absolute numbers with percentages, and quantitative variables were presented as means with standard deviations.

## 3. Results

A total of 75 patients were included in this study, with their demographic and burn-related characteristics summarized in Table 1. The mean age of the patients was 48.6 ± 18.9 years. The majority of the study population was male (69.3%), and the mean affected body surface area (BSA) was 4.6 ± 2.9%. Of the patients, 18.7% had third-degree burn wounds, and 10.7% had an acute inhalation injury. The extent of enzymatic debridement with NexoBrid^®^ varied, with most patients (57.3%) receiving debridement over less than 3% of their BSA. The mean length of hospital stay was 11.5 ± 13.0 days, and the mortality rate was 4.0%. Data from the three non-survivors were included in all statistical analyses, except for length of hospital stay. Two patients died from pneumonia-associated septic multiorgan failure following inhalation trauma confirmed by bronchoscopy. One patient with significant pre-existing conditions (including liver cirrhosis and esophageal varices) died due to impaired gas exchange and early multiorgan failure.

The comparison of clinical and laboratory parameters before and after NexoBrid^®^ application is presented in Table 2. Pain levels, assessed using the numerical rating scale (NRS), showed a slight increase after NexoBrid^®^ treatment (2.3 ± 1.9 vs. 2.7 ± 2.2), though this change was not statistically significant (*p* = 0.340). A significant increase in body temperature was observed following treatment (36.8 ± 0.5 °C before vs. 37.0 ± 0.7 °C after, *p* = 0.018). Hemoglobin levels significantly decreased after NexoBrid^®^ application (13.3 ± 1.6 g/dL before vs. 12.6 ± 1.7 g/dL after, *p* < 0.001), while the prothrombin time ratio (% of normal) significantly decreased (82.0 ± 13.3% before vs. 76.8 ± 14.9% after, *p* = 0.002), and the C-reactive protein (CRP) levels significantly increased (4.8 ± 5.8 mg/dL before vs. 10.8 ± 7.2 mg/dL after, *p* < 0.001). Other parameters, including leukocyte count, activated partial thromboplastin time, sodium, potassium, and creatinine levels, did not show statistically significant differences before and after treatment.

Further analysis comparing patients with ≤3% and >3% BSA exposure to NexoBrid^®^ is summarized in Table 3. Apart from a slightly higher mean body temperature in the higher-exposure group (*p* = 0.036), no statistically significant differences were observed between the groups. Pain levels tended to be lower in the higher-exposure group, but this did not reach significance. The laboratory parameters, including leukocyte count, hemoglobin, coagulation values, electrolytes, and inflammatory markers, remained comparable between the two groups, indicating no clear dose-dependent systemic effect.

## 4. Discussion

The primary aim of this study was to systematically investigate the systemic effects of enzymatic debridement using NexoBrid^®^ in patients with burn injuries. Our analysis focused on evaluating changes in systemic inflammatory markers, coagulation parameters, and other relevant clinical parameters before and after NexoBrid^®^ application. The main findings indicate that while NexoBrid^®^ is effective in enzymatic debridement, it is associated with significant increases in body temperature and C-reactive protein levels, suggesting mild systemic inflammatory changes. Additionally, we observed a significant decrease in the prothrombin time ratio (% of normal) post-treatment, which may indicate an effect on coagulation, potentially also influenced by minor bleeding during or after NexoBrid^®^ exposure. However, other parameters such as leukocyte count, electrolyte levels, and creatinine showed no significant changes following NexoBrid^®^ treatment, although a significant decrease in hemoglobin levels was observed. These results provide valuable insights into the broader systemic implications of NexoBrid^®^ use in burn care.

The notable decrease in the prothrombin time ratio (% of normal) suggests that NexoBrid^®^ potentially had effects on coagulation. These results align with earlier reports highlighting the complex interaction between burn injuries, enzymatic debridement, and coagulation pathways [17,21,22]. Bulla et al. (2025) reported coagulation alterations, most commonly elevated prothrombin time, in 40% of evaluated patients after enzymatic debridement [23]. Furthermore, 7% of patients required blood transfusions, with a significant association between transfusion need and the treated TBSA, which aligns with our observation of a post-treatment hemoglobin decrease. Notably, Hasham et al. (2023) documented cases where NexoBrid^®^ influenced coagulation, underscoring the need for careful monitoring in patients with pre-existing clotting disorders [17]. On the other hand, the study by Pfister et al. (2023) provides additional context to our findings, demonstrating that patients who underwent enzymatic debridement with bromelain-based agents did not show significant differences in key coagulation parameters, such as the international normalized ratio and aPTT, compared to those who received surgical debridement [21]. Interestingly, their study also reported elevated Factor V levels in patients treated with enzymatic debridement. However, our study found a significant difference in the prothrombin time ratio (% of normal) before and after NexoBrid^®^ application, indicating that the impact of enzymatic debridement on coagulation may vary depending on specific clinical conditions or patient characteristics. This discrepancy suggests that the effects of enzymatic debridement on coagulation remain an open question and warrant further investigation, particularly in larger, multicenter studies.

While our study identified statistically significant increases in CRP levels and body temperature following NexoBrid^®^ application, it is important to critically assess their clinical relevance. The absolute change in CRP, though statistically significant, was relatively mild and may not reflect a clinically significant systemic inflammatory response in this patient population. It should also be noted that the observed laboratory and clinical changes did not meet the clinical criteria for Systemic Inflammatory Response Syndrome (SIRS) and should not be equated with this more severe systemic condition. Furthermore, the average body temperature remained within the normothermic range, and the leukocyte counts showed no significant change. Taken together, these findings support the interpretation that NexoBrid^®^ does not induce a clinically relevant systemic inflammatory response in patients with ≤10% TBSA burns. Importantly, these changes were self-limiting and not associated with confirmed infections or systemic complications, reinforcing the interpretation that such alterations may represent localized and transient inflammatory responses rather than systemic sepsis or coagulopathy. These considerations are further supported by Bulla et al. (2025), who reported early post-debridement fever in 14% of patients [23]. Interestingly, fever occurred more frequently in younger individuals and was associated with an increased risk of subsequent wound infection (OR ≈ 2.5). These findings suggest that early fever, while not necessarily indicative of systemic infection, may warrant closer surveillance in selected patients. Our findings are partially consistent with those reported by Pfister et al. (2023), who also noted an increase in CRP levels after enzymatic debridement [21]. Unlike our study, they did not find a statistically significant difference in body temperature following enzymatic debridement treatment [21]. Moreover, Hofmaenner et al. (2021) investigated the systemic inflammatory effects of NexoBrid^®^ in patients undergoing off-label extensive debridement (greater than 15% body surface area, BSA) compared to regular use [24]. They found no significant changes in inflammatory markers after NexoBrid^®^, including CRP and leukocytes, between the two groups, suggesting that extensive use of NexoBrid^®^ does not necessarily lead to a heightened systemic inflammatory response. Similarly, our study found that while body temperature was significantly higher in patients with a higher degree of BSA exposure, other inflammatory markers such as CRP levels did not show a proportional increase, indicating that the inflammatory effects may not be directly related to the treated area. Finally, despite the inflammatory response observed in our study, Deplazes et al. (2023) showed in their study that enzymatic debridement does not increase susceptibility to systemic infections such as bacteremia [25]. These differences across studies may be due to variations in study design, patient populations, and the extent of BSA treated, suggesting that further research is needed to fully understand the systemic effects of enzymatic debridement in burn care.

This study stands out as the largest individual investigation into the systemic effects of enzymatic debridement using NexoBrid^®^ in burn patients, with a sample size of 75 cases. This large cohort enhances the reliability of our observations and allows for a detailed comparison of the outcomes in patients with less versus more NexoBrid^®^ exposure within the ≤10% TBSA group. Importantly, our findings suggest that a higher degree of NexoBrid^®^ application does not necessarily lead to more pronounced systemic changes in this patient population. This insight is particularly relevant for clinical decision-making in patients with smaller burn injuries (≤10% TBSA), where concerns about systemic inflammation and coagulopathy remain significant.

However, our study has several limitations. The retrospective and pre–post observational design, without a contemporaneous control group, limits our ability to establish causality regarding the effects of NexoBrid^®^. Although we aimed to minimize confounding by focusing on ≤10% TBSA burns, residual effects from the burn injury itself remain a potential influence. The lack of a defined control group, as well as the absence of extended laboratory or clinical follow-up data (e.g., at three- or five-days post-intervention), restricts our conclusions to the immediate pre–post changes observed in this specific cohort.

Despite these limitations, our findings contribute important descriptive observations about mild systemic changes following NexoBrid^®^ use in patients with smaller burns. These insights may help to identify patient subgroups at risk of systemic reactions and could inform more individualized treatment decisions in the future. While the observational nature of our study limits causal interpretation, our findings provide relevant insights that may inform clinical practice and guide future research. Future prospective, controlled studies are necessary to more precisely isolate the effects of NexoBrid^®^ from the natural systemic responses to burn injuries.

## 5. Conclusions

While we observed statistically significant changes in body temperature, hemoglobin, and C-reactive protein (CRP) levels following NexoBrid^®^ application in ≤10% TBSA burns, these changes were mild and not clinically significant. This suggests that although mild systemic inflammatory changes occur with NexoBrid^®^ use, they do not amount to a clinically relevant systemic inflammatory response. This underlines the importance of individualized monitoring in burn patients and supports the safe use of NexoBrid^®^ as part of a personalized burn treatment approach in selected cases. Clinically, these observations suggest that NexoBrid^®^ can be safely used in patients with smaller burn injuries (≤10% TBSA), but in order to make definitive conclusions about its safety and systemic effects—particularly in more extensive burns—further controlled studies are required. Notably, the most prominent laboratory change observed was a significant decrease in hemoglobin levels, which should be considered when evaluating the systemic safety of enzymatic debridement, especially in vulnerable patient populations.

## Figures and Tables

**Table 1:** Patient demographics and burn characteristics.

Parameter	Study Group (*n* = 75)
Age, years	48.6 ± 18.9
Gender	
Male	52 (69.3%)
Female	23 (30.7%)
Affected body surface area (BSA), %	4.6 ± 2.9
Third-degree burn wounds	
Yes	14 (18.7%)
No	61 (81.3%)
Acute inhalation injury	
Yes	8 (10.7%)
No	67 (89.3%)
Extent of enzymatic debridement, % of BSA	
≤3%	43 (57.3%)
>3%	32 (42.7%)
Length of hospital stay, days	11.5 ± 13.0
Mortality	
Survivors	72 (96.0%)
Non-survivors	3 (4.0%)

**Table 2:** Clinical and laboratory parameters before and after NexoBrid^®^ application.

Parameter	Before NexoBrid^®^	After NexoBrid^®^	*p*-Value
Pain, numerical rating scale	2.3 ± 1.9	2.7 ± 2.2	0.340
Body temperature, °C	36.8 ± 0.5	37.0 ± 0.7	**0.018**
Leukocyte count, per mm^3^	11.8 ± 5.0	11.8 ± 5.9	0.927
Hemoglobin, g/dL	13.3 ± 1.6	12.6 ± 1.7	**<0.001**
Prothrombin time ratio, % of normal	82.0 ± 13.3	76.8 ± 14.9	**0.002**
Activated partial thromboplastin time, seconds	30.0 ± 10.3	29.5 ± 7.5	0.522
Sodium, mmol/L	135.7 ± 2.7	139.8 ± 3.1	0.299
Potassium, mmol/L	4.1 ± 0.3	4.1 ± 0.4	0.945
Creatinine, mg/dL	1.0 ± 0.4	1.1 ± 0.5	0.159
C-reactive protein, mg/dL	4.8 ± 5.8	10.8 ± 7.2	**<0.001**

**Table 3:** Comparison of clinical and laboratory parameters after NexoBrid^®^ application based on extent of BSA exposure to NexoBrid^®^.

Parameters After NexoBrid^®^	Limited BSA Exposure (*n* = 43)	Higher BSA Exposure (*n* = 32)	*p*-Value
Pain, numerical rating scale	3.1 ± 2.2	1.7 ± 2.0	0.062
Body temperature, °C	36.9 ± 0.6	37.3 ± 0.8	**0.036**
Leukocyte count, per mm^3^	12.5 ± 7.4	11.2 ± 4.4	0.551
Hemoglobin, g/dL	13.1 ± 1.3	12.1 ± 1.8	0.106
Prothrombin time ratio, % of normal	82.1 ± 14.8	73.1 ± 14.3	0.095
Activated partial thromboplastin time, seconds	26.6 ± 7.7	31.5 ± 6.9	0.065
Sodium, mmol/L	139.9 ± 2.5	139.7 ± 3.5	0.904
Potassium, mmol/L	4.0 ± 0.4	4.1 ± 0.3	0.352
Creatinine, mg/dL	0.9 ± 0.3	1.2 ± 0.7	0.262
C-reactive protein, mg/dL	9.9 ± 7.8	13.0 ± 7.9	0.834

## Data Availability

The data that support the findings of this study are available upon reasonable request from the corresponding author [D.B.].
